# How Polyomaviruses Exploit the ERAD Machinery to Cause Infection

**DOI:** 10.3390/v8090242

**Published:** 2016-08-29

**Authors:** Allison Dupzyk, Billy Tsai

**Affiliations:** 1 Department of Microbiology and Immunology, University of Michigan Medical School, 1150 West Medical Center Drive, Ann Arbor, MI 48109, USA; adupzyk@umich.edu; 2 Department of Cell and Developmental Biology, University of Michigan Medical School, 109 Zina Pitcher Place, BSRB 3043, Ann Arbor, MI 48109, USA

**Keywords:** polyomavirus, SV40, ERAD, protein aggregation, membrane penetration

## Abstract

To infect cells, polyomavirus (PyV) traffics from the cell surface to the endoplasmic reticulum (ER) where it hijacks elements of the ER-associated degradation (ERAD) machinery to penetrate the ER membrane and reach the cytosol. From the cytosol, the virus transports to the nucleus, enabling transcription and replication of the viral genome that leads to lytic infection or cellular transformation. How PyV exploits the ERAD machinery to cross the ER membrane and access the cytosol, a decisive infection step, remains enigmatic. However, recent studies have slowly unraveled many aspects of this process. These emerging insights should advance our efforts to develop more effective therapies against PyV-induced human diseases.

## 1. Introduction

Polyomaviruses (PyVs) are small non-enveloped DNA tumor viruses belonging to the *Polyomaviridae* family. The first two human PyVs, JC and BK PyV, were discovered in 1971 [1,2]. Since then, an additional 11 human PyVs have been uncovered [3,4,5], including some more prominent viruses such as the Merkel cell PyV, the causative agent of an aggressive skin cancer called Merkel cell carcinoma [6]. PyVs are highly prevalent in the human population, with some such as the BK PyV estimated to infect up to approximately 90% of the human population [3,7,8,9]. Although PyV infections are generally benign in healthy immunocompetent individuals, they pose a significant threat to immunocompromised patients [9]. For instance, BK PyV, the causative agent of PyV-associated nephropathy [8], becomes problematic in transplant patients. Because no therapies currently exist for PyV-associated nephropathy, treatment requires reducing immunosuppressants, which often leads to graft rejection. As another example, JC PyV is a neurotropic virus that causes progressive multifocal leukoencephalopathy (PML), a demyelinating disease of the central nervous system [10]. In immunocompromised patients such as in patients infected by human immunodeficiency virus and suffering acquired immune deficiency syndrome (HIV/AIDS), JC PyV-induced PML is rather common. Despite the fact that PyVs were discovered 45 years ago, effective treatments are currently lacking. However, recent advances in PyV research have made therapeutic development a realistic possibility. 

**Figure 1:**
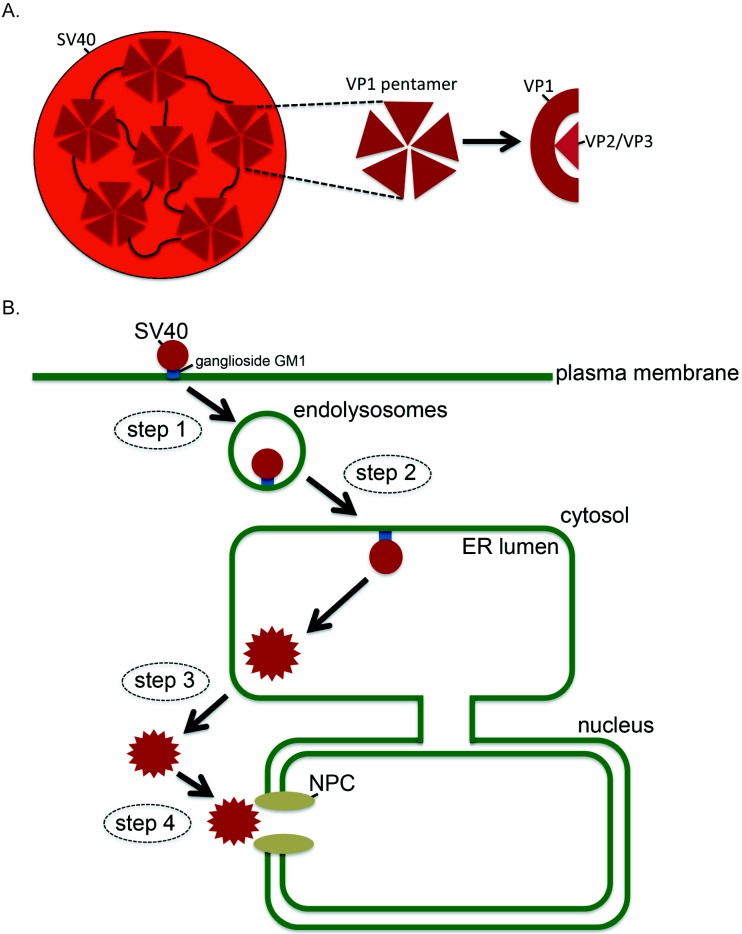
Simian virus 40 (SV40) structure and entry pathway. (**A**) SV40 consists of 360 VP1 copies arranged as 72 pentamers, which are localized on the viral surface. The pentamers are stabilized by disulfide bonds, as well as by interactions between the VP1 carboxy-terminus, which invades a neighboring pentamer (black curved lines). VP1 also binds to the underlying internal hydrophobic proteins VP2 and VP3. (**B**) To infect cells, SV40 interacts with the glycolipid receptor ganglioside GM1 on the plasma membrane, internalizes, and traffics to the endolysosomes (step 1). The virus then targets to the endoplasmic reticulum (ER) using a lipid-sorting mechanism (step 2), from where it crosses the ER membrane to access the cytosol (step 3). Upon entering the cytosol, SV40 mobilizes into the nucleus (step 4), where ensuing transcription and replication of the viral genome causes lytic infection or cellular transformation. NPC: nuclear pore complex.

In this review, we will discuss host entry of the PyV simian virus 40 (SV40) due to the wealth of available information; where relevant, other PyVs will also be described. SV40 is the archetype PyV, displaying both genetic and structural similarity to human PyVs. Structurally, SV40 contains a circular doubled-stranded DNA genome of approximately five kilobases that encodes seven genes, three structural genes called *VP1*, *VP2*, *VP3*, and four non-structural genes called *VP4*, *large T antigen*, *small T antigen*, and *agno protein* [3,5,9,11]. As a non-enveloped virus, SV40 lacks a surrounding envelope and instead contains a protein capsid composed of 360 VP1 copies arranged as 72 pentamers that are displayed on the viral surface (Figure 1A). The pentamers are stabilized by disulfide bonds, as well as by interactions between the VP1 carboxy-terminus, which invades a neighboring pentamer [12,13]. VP1 also associates with the underlying internal hydrophobic proteins VP2 and VP3, which along with VP1, bind to the genome [14]. When fully assembled, SV40 is approximately 45–50 nm in diameter [5,9,12].

To infect cells, SV40 first binds to the glycolipid receptor ganglioside GM1 on the cell surface [15,16,17], becomes internalized, and traffics to the endolysosomes (Figure 1B, step 1; [18]). The virus then sorts to the endoplasmic reticulum (ER) using a lipid-directed mechanism (Figure 1B, step 2; [15,19,20]), from where it ejects across the ER membrane to reach the cytosol (Figure 1B, step 3). Upon reaching the cytosol, SV40 mobilizes to the nucleus (Figure 1B, step 4), where ensuing transcription and replication of the viral genome causes lytic infection or cellular transformation [21,22]. Although SV40 ER-to-cytosol membrane penetration—a decisive infection step—is not entirely understood, key concepts and players involved in this pathway are slowly being unraveled [23]. The emerging central principle posits that SV40 hijacks elements of a major cellular protein quality control pathway called ER-associated degradation (ERAD) during its ER membrane penetration. 

## 2. What is ERAD?

**Figure 2:**
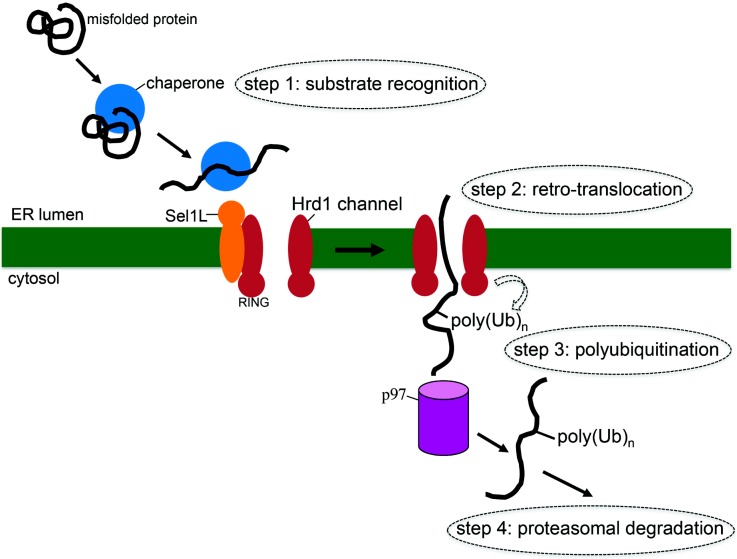
ER-associated degradation (ERAD) pathway. ERAD is an ER quality control pathway that identifies and triages misfolded ER proteins. In the first step, a misfolded protein is recognized by ER-resident chaperones, which target the misfolded client to the retro-translocation machinery on the ER membrane (step 1). Next the misfolded client is retro-translocated across the ER membrane by crossing the retro-translocation channel (step 2); a major component of this channel is the Sel1L-Hrd1 membrane complex. When the client emerges into the cytosol, it is ubiquitinated by the catalytic domain of Hrd1 that faces the cytosol, eventually resulting in polyubiquitinaton of the substrate (step 3). In the final step, the client is extracted into the cytosol by p97 (and its cofactors), and delivered to the proteasome for degradation (step 4). Sel1L: protein sel-1 homolog 1; Hrd1: E3 ubiquitin-protein ligase synoviolin; Poly(Ub)_n_: polyubiquitin chain; RING: Really Interesting New Gene finger domain.

The ER is thought to be an oxidative membrane-bound organelle that specializes in protein folding. These ER-folding clients often represent proteins that are destined for secretion along the classical secretory pathway, which is physically connected to the ER. During co-translational translocation of nascent polypeptide chains into the ER, the nascent polypeptide is transported across the ER membrane by crossing the Sec61 translocation channel [24]. In the ER, numerous ER luminal chaperones, post-translational modifiers, and folding catalysts assist in the protein folding process. For instance, carbohydrates are appended to a nascent polypeptide by the oligosaccharyl-transferase (OST) complex [25], while disulfide bonds of the polypeptide are formed and rearranged by members of the protein disulfide isomerase (PDI) family [26]. Moreover, to prevent a polypeptide chain from aggregation and render it soluble, molecular chaperones such as the 70 kDa heat shock protein (Hsc70) ATPase binding immunoglobulin protein (BiP) are recruited to the folding intermediate [27]. These coordinated efforts enable the polypeptide to attain its native configuration and proper oligomeric state. Once formed and assembled, a folded polypeptide is packaged into coat protein complex II (COPII) vesicles and exits the ER en route for secretion. Because approximately one third of all mammalian genes encode proteins that are translocated into the ER, it is not surprising that the ER maintains a quality control system that prevents the generation of misfolded or aggregated ER proteins, and has the capacity to actively remove these aberrant species should they form. Over the past two decades, ERAD has been identified as the key ER quality control process dedicated to the removal of misfolded ER proteins [28,29]. During ERAD, misfolded ER clients are recognized and ejected into the cytosol where they are in turn degraded by the ubiquitin-dependent proteasome machinery. Conceptually, the quality control process can be divided into four distinct steps: substrate recognition, retro-translocation across the ER membrane, substrate polyubiquitination, and proteasomal degradation (Figure 2).

In the first step, a misfolded ER luminal (or transmembrane) client is identified (Figure 2, step 1). Identification is accomplished through recognition of improper carbohydrate residues or erroneous disulfide bond pairing, resulting in aberrant exposure of hydrophobic patches or oligomer formation in a misfolded polypeptide. ER-resident factors including protein disulfide isomerase (PDI) family members [26], BiP [30,31], and enzymes involved in carbohydrate recognition and processing such as ER class I α-mannosidase (ER ManI) [32], ER degradation-enhancing α-mannosidase-like (EDEM)1/3 [33], and osteosarcoma amplified 9/(OS-9/XTP3) [34,35,36], all serve critical roles during this initial committed step of the ERAD pathway. 

Once identified, the misfolded polypeptide is targeted to a protein-conducting channel that retro-translocates the substrate from the ER into the cytosol (Figure 2, step 2). Although pinpointing the identity of this “retrotranslocon” remains a major challenge, recent studies suggest that the multi-pass ER transmembrane protein called E3 ubiquitin-protein ligase Hrd1 (synoviolin) is a strong candidate [37,38,39]; other components such as the Hrd1-interacting partners called Derlins have also been implicated [40,41]. Before a misfolded client engages Hrd1, it is first delivered to another Hrd1-associated membrane factor called protein sel-1 homolog 1 (Sel1L), which is generally thought to act as a substrate acceptor during ERAD [42]. Sel1L in turn “hands off” a client to Hrd1, preparing the client for transport across the ER membrane. It is important to note that Hrd1 itself is an E3 ubiquitin ligase that displays its so-called catalytic Really Interesting New Gene (RING)-finger domain towards the cytosol. This is an important structural feature because ubiquitination plays at least two distinct functions during ERAD: Hrd1-triggered autoubiquitination of its RING-finger domain appears to gate its channel activity [39], while Hrd1’s ability to facilitate substrate ubiquitination plays an essential role in substrate extraction from the ER membrane (see below).

During the third ERAD step (Figure 2, step 3), Hrd1 operates in conjunction with E2 conjugating enzymes such as ubiquitin-conjugating enzyme E2, G2 (Ube2g2) and E2, J1 (Ube2j1) [43,44] to promote ubiquitination of lysine residues in a misfolded client that has emerged into the cytosol; additional rounds of ubiquitination subsequently generate a polyubiquitin chain. There is evidence that serine, threonine, and cysteine residues can also serve as the ubiquitination sites [45]. Regardless, polyubiquitination is thought to prevent “back-sliding” of the client into the ER lumen, thereby favoring client release into the cytosol. Passive release of the polyubiquitinated substrate into the cytosol does not occur, but instead is catalyzed by cytosolic chaperones that actively extract the substrate. This is primarily accomplished by the ATPases Associated with diverse cellular Activities (AAA) ATPase p97, which functions in a concerted manner with its co-factors ubiquitin fusion degradation 1 (Ufd1) and nuclear protein localization protein 4 (Npl4) [46]. 

In the last step of ERAD (Figure 2, step 4), the polyubiquitinated substrate is delivered to the proteasome for destruction. Delivery to the proteasome likely involves a recently discovered holdase complex consisting of BCL2 associated athanogene 6 (Bag6), ubiquitin-like protein 4A (Ubl4a), transmembrane domain recognition complex 35 (Trc35), and small glutamine-rich tetratricopeptide repeat-containing protein alpha (SGTA) that prevents the misfolded client from aggregation in the cytosol [47,48]. Upon reaching the proteasome, deubiquitination and unfolding activities associated with the proteasome machinery remove the polyubiquitin chain and unfold the substrate. These reactions allow a misfolded client to properly thread through the central chamber of the proteasome so that hydrolysis of the polypeptide chain can ensue [49].

While ERAD’s ability to rectify the protein-misfolding problem is essential to maintain overall cellular protein homeostasis (proteostasis), its central feature—presence of a physical conduit between the ER luminal and cytosolic space—can be exploited during pathogen-host interactions. And indeed, nowhere is this more evident than during ER-to-cytosol membrane penetration by SV40. 

## 3. How SV40 Hijacks Elements of ERAD during ER Membrane Transport

### 3.1. ER Luminal Events 

**Figure 3:**
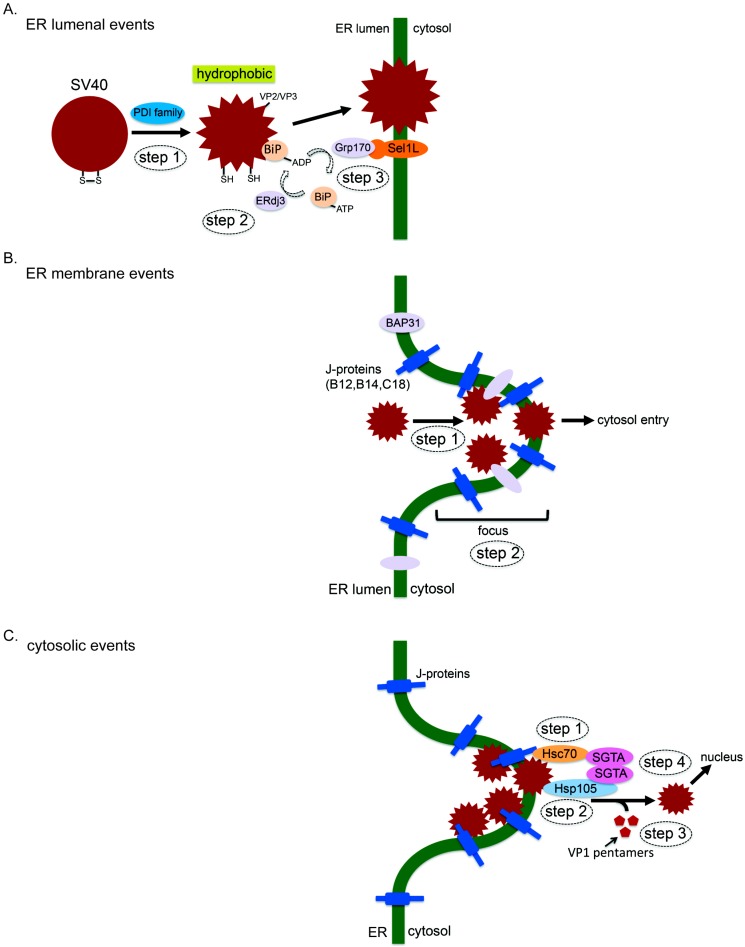
ER-to-cytosol membrane penetration of SV40. Penetration across the ER membrane to reach the cytosol is a decisive SV40 infection step. This process can be conceptually divided into ER lumenal, membrane, and cytosolic events. (**A**) During ER lumenal events, protein disulfide isomerase (PDI) family members impart conformational changes to SV40, generating a hydrophobic viral particle by exposing its VP2 and VP3 hydrophobic proteins (step 1). This hydrophobic virus is maintained in a soluble state by interacting with ADP-binding immunoglobulin protein (BiP), which is formed by the action of the J-protein ERdj3 (step 2). When the SV40-BiP complex is proximal to the ER membrane, the nucleotide exchange factor glucose-regulated protein 170 kDa (Grp170) induces nucleotide exchange of BiP, generating ATP-BiP that releases SV40 (step 3). The hydrophobic SV40 in turn binds to and integrates into the ER membrane to initiate membrane transport. (**B**) During ER membrane events, the membrane-embedded SV40 binds to the B-cell receptor-associated protein 31 (BAP31) membrane protein (step 1), a step thought to stabilize the viral structural integrity. Concomitant with this step, SV40 also induces the lateral reorganization of different ER membrane proteins (including BAP31 and the J-proteins B12, B14, and C18) to form discrete puncta called foci (step 2)—the foci structures are believed to represent the cytosol entry sites. How this virus induces foci formation is not entirely understood. (**C**) During the cytosolic events, the J-proteins B12/B14/C18 recruit a cytosolic complex composed of 70 kDa heat shock protein (Hsc70), human heat shock protein 105 kDa (Hsp105), and small glutamine-rich tetratricopeptide repeat-containing protein alpha (SGTA) that extracts SV40 into the cytosol. The J-proteins first convert Hsc70 to ADP-Hsc70, allowing this chaperone to bind to SV40. The nucleotide exchange factor Hsp105 changes ADP-Hsc70 to ATP-Hsc70, which releases SV40 from Hsc70. Because Hsp105 is also a bonafide chaperone, it captures SV40 once the virus is released from Hsc70. Iterative cycles of Hsc70-Hsp105 binding to and release from SV40 is thought to extract SV40 into the cytosol. Hsp105 can also disassemble the virus, a reaction that may be coupled to the extraction process. SGTA’s precise function is unclear, but can either regulate Hsc70’s ability to engage SV40, bring Hsc70 and Hsp105 in proximity due to its ability to dimerize, or catalyze an event post ER membrane penetration such as in facilitating cytosol-to-nucleus transport. SH: hydrosulfide radical.

After trafficking to the ER, SV40 is thought to disguise as a “misfolded” substrate, co-opting components of the ERAD machinery in order to penetrate the ER membrane and reach the cytosol. To do so, it first undergoes conformational changes that partially uncoat the virus. This conformational change generates a hydrophobic viral particle that binds to and inserts into the ER membrane, a step required for successful membrane transport. Multiple PDI family members impart SV40 structural alterations in the ER, including PDI, ERp57, and ERdj5 (Figure 3A, step 1; [50,51,52]); there is evidence that ERdj5 also executes an important role during BK PyV infection [52]. In the case of the murine PyV, another PDI family member called ERp29 was found to locally unfold the VP1 carboxy-terminal arm [53,54], a reaction that in conjunction with PDI and ERp57 [51,55] generates a hydrophobic virus by exposing the internal hydrophobic proteins VP2 and VP3 [56,57]. Of note, the PDI-ERp57-ERp29 triad has also been reported to act on JC PyV during infection [58].

Uncoating of SV40 (and other PyVs) by PDI family members exposes the underlying hydrophobic proteins VP2 and VP3. While the newly-generated hydrophobic viral particle can engage and integrate into the hydrophobic ER membrane, this virus is also prone to aggregation that can result from non-productive self interactions via previously hidden viral hydrophobic regions. To prevent aggregation, BiP is recruited to the virus [59,60], similar to its role in avoiding aggregation of a misfolded client during ERAD [27]. BiP’s ability to engage SV40 (and other cellular substrates) is strictly dependent on its nucleotide-bound states [61]. In the adenosine diphosphate (ADP)-bound form, BiP has a high affinity for its substrate, while adenosine triphosphate (ATP)-BiP displays a low substrate-binding affinity. J-proteins activate BiP’s intrinsic ATPase activity, converting ATP-BiP to ADP-BiP. By contrast, nucleotide exchange factors (NEFs) release ADP from ADP-BiP, enabling ATP to re-engage BiP and generate ATP-BiP. In this context, the ER-resident J-protein ERdj3 (also called B11) was found to promote SV40 ER membrane transport by stimulating SV40-BiP interaction (Figure 3A, step 2; [60]), presumably after hydrophobic proteins VP2 and VP3 are exposed. When the SV40-BiP complex is proximal to the luminal surface of the ER membrane, the virus must be released from BiP in order to initiate ER membrane transport. Of the two established ER luminal NEFs, only glucose-regulated protein 170 kDa (Grp170), but not Sil1, promotes SV40 release from BiP in order to prime the virus for membrane penetration (Figure 3A, step 3; [62]). Grp170 was also shown to bind directly to the Hrd1 adapter Sel1L [63]. This positions Grp170 next to the ER membrane, suggesting that SV40 release from BiP occurs proximal to the membrane. Such a scenario raises the possibility that release of the virus from BiP is coupled to membrane transport. Interestingly, only Sel1L [50] but not Hrd1 [59] plays a role in SV40 and JC PyV infection [58]. Why this is the case is unclear, but there is the possibility that Sel1L might operate independent of Hrd1 in some instances. For instance, there may exist a pool of Sel1L that does not bind to Hrd1. Instead, this Sel1L pool might recruit previously uncharacterized ER (or possibly cytosolic) proteins that promote PyV infection. Perhaps one rational approach to tackle this possibility is to isolate the Sel1L pool that does not associate with Hrd1, and carefully dissect potential Sel1L-binding partners using this pool.

While not identical for all PyV family members, a clear theme has come into focus for ER luminal events initiating the membrane transport process. Specifically, upon reaching the ER from the plasma membrane, the virus initially undergoes conformational changes induced by ERAD factors such as the PDI family members—these reactions expose the inner hydrophobic VP2 and VP3 proteins and generate a hydrophobic viral particle. The hydrophobic virus is prevented from aggregation by the recruitment of ERAD molecular chaperones such as BiP. In the final phase, the hydrophobic virus disengages from the molecular chaperone, binds to and inserts into the ER membrane, and is now primed for penetration across the lipid bilayer. 

### 3.2. ER Membrane Events 

When the hydrophobic SV40 particle inserts into the ER membrane, the amino-terminal region of the exposed VP2 protein binds to an ER membrane protein called B-cell receptor-associated protein 31 (BAP31) (Figure 3B, step 1; [59]), which is thought to stabilize the membrane-embedded virus. Additional ER membrane components such as the Derlins have also been reported to mediate ER-to-cytosol transport of SV40 [50], as well as the murine PyV [64], and BK PyV [65]. However, the precise molecular contribution of Derlins to the membrane penetration event is unclear.

In an unbiased RNA interference (RNAi) screen, three ER transmembrane J-proteins (B12, B14, and C18) were found to be essential in promoting SV40 and BK PyV infection [60]. Of these J-proteins, B12 and B14 have been previously implicated in the ERAD process [66,67,68]. Not surprisingly, due to their localization to the ER membrane, B12, B14, and C18 regulate the decisive virus ER-to-cytosol membrane transport step [60,69,70]. Because these J-proteins display their catalytic J-domain towards the cytosol, part of their mechanism of action likely involves the recruitment of cytosolic Hsc70 and associated co-chaperones used to extract the virus into the cytosol (see below). Interestingly, using a knockdown-rescue approach, all three J-proteins were shown to exert non-redundant roles during SV40 ER membrane penetration [70]. These results suggest that each J-protein imparts a unique function within the viral membrane transport pathway. One possibility is that an individual J-protein binds to a distinct set of luminal, membrane, or cytosolic partners that are all necessary to support the membrane transport process.

An outstanding question is whether there are specific regions within the vast ER membrane, which is composed of a complicated network of sheets and tubules [71], that serve as selective membrane penetration sites for the virus. To address this question, we and others reported that many of the ER membrane proteins that promote SV40 ER membrane transport, including BAP31 (and the related BAP29), B12, B14, and C18, reorganize into distinct subdomains within the ER membrane called foci during SV40 infection (Figure 3B, step 2; [59,69,70,72]); SV40 itself also accumulates in these virus-induced foci [59,69,70,72]. Importantly, the VP2/VP3 exposed, membrane penetration-competent form of SV40 is found predominantly in these punctate structures [70]. These collective findings suggest that virus-triggered foci represent viral cytosol entry sites from the ER. Consistent with this idea, the rate of foci formation was found to temporally parallel SV40 cytosol arrival from the ER [69], SV40 mutants which cannot transfer across the ER membrane to reach the cytosol also fail to trigger foci formation [70], and impairing SV40 release into the cytosol traps SV40 in the foci leading to expansion of the foci structure [72]. While these are compelling data to support the notion that SV40 possesses a unique ability to construct specific penetration sites on the ER membrane, the precise molecular mechanism by which the viral particle stimulates foci formation remains unclear. For instance, assuming that individual components of the foci structure must reorganize laterally within the ER lipid bilayer to generate the foci structure, how does SV40 exploit cellular mechanical forces to accomplish this difficult feat? Are these forces provided by additional ER luminal, membrane, or cytosolic factors during SV40 infection? Clearly, clarifying the nature of the virus-induced foci, as well as elucidating its precise physiologic functions, deserves more attention.

### 3.3. Cytosolic Events

Although SV40 is partially uncoated when it penetrates the ER membrane, the size of these viral particles remains relatively large, with reports ranging from approximately 35 nm [59] up to 45 nm [52,73] in diameter. Thus it is unlikely that the membrane-inserted SV40 passively slips through the ER lipid bilayer to reach the cytosol. Instead, we postulate that a cytosolic extraction machinery “pulls” the viral particle into the cytosol. During ERAD, p97 normally provides the primary driving force to extract a misfolded client from the ER into the cytosol [46]. However, this cytosolic ATPase is not involved in SV40 infection [59]. Motivated by this observation, we used a classical biochemical approach to identify the putative cytosolic extraction machinery, guided by the basic premise that the membrane-bound J-proteins B12, B14, and C18 would recruit such a machinery. These efforts pinpointed a cytosolic complex composed of Hsc70, human heat shock protein 105 kDa (Hsp105), and SGTA that plays an essential role in ejecting SV40 into the cytosol [69,72]. Individual components of the Hsc70-Hsp105-SGTA complex are known to play a role during the cytosolic phase of ERAD [47,48,74]. While the precise mechanism by which this ternary complex coordinately extracts SV40 into the cytosol is not fully understood, a working model has nonetheless emerged. 

Via the action of the membrane-bound J proteins, Hsc70 is converted to the high-affinity ADP-Hsc70 state. This enables Hsc70 to initially bind to the membrane-embedded SV40 (Figure 3C, step 1). As a NEF, Hsp105 then induces nucleotide exchange of Hsc70, generating ATP-Hsc70 that releases the viral particle. In addition to acting as a NEF, Hsp105 is also an extended member of the Hsc70 ATPase superfamily that harbors a bonafide chaperone activity [75,76]. Thus Hsp105 is able to capture SV40 once the virus disengages from Hsc70 (Figure 3C, step 2). When SV40 is released from Hsp105, Hsc70 in turn re-binds the virus. Iterative rounds of Hsc70-Hsp105 binding to and release from SV40 are thought to provide the major driving energy to extract the viral particle from the ER membrane. Strikingly, Hsp105 was reported to have a powerful disaggregation activity when it operates in concert with Hsc70 and a J-protein [77,78,79]. In line with this finding, our analyses demonstrate that Hsp105 can disaggregate the virus by removing the VP1 pentamers (Figure 3C, step 3; [72]). Thus, Hsp105 in conjunction with Hsc70 and the membrane-bound J-proteins could act coordinately to disassemble the virus, generating a smaller SV40 species that facilitates viral release into the cytosol. The discovery that a disaggregation activity is used to drive cytosolic release of a virus from the ER immediately suggests that such an activity would be used during canonical ERAD. In this regard, many misfolded ER membrane proteins have been reported to aggregate [80], raising the intriguing scenario that the Hsp105-dependent disaggregation activity can be harnessed to disaggregate these clients prior to their release into the cytosol. Future experiments are needed to clarify this possibility.

The final member of the cytosolic extraction complex is SGTA [69,81]. This factor is a promiscuous cytosolic protein that plays diverse roles in many cellular pathways. For instance, SGTA exerts a critical function during ERAD, acting as a component of the Bag6-Ubl4A-Trc35-SGTA holdase complex that couples misfolded clients release from the ER and delivery to the proteasome (Figure 2; [47,48]). Additionally, SGTA catalyzes an important step in the guided entry of tail-anchored proteins (GET) pathway, which inserts tail-anchored (TA) proteins from the cytosol into the ER membrane [82,83]. Conceptually, SGTA acts in a completely opposite manner within the GET pathway when compared to its role during SV40 extraction: in the GET pathway, SGTA delivers a hydrophobic protein from the cytosol to the ER, while it extracts a hydrophobic protein complex (SV40) from the ER into the cytosol during SV40 infection. 

Precisely how SGTA assists Hsc70 and Hsp105 in ejecting SV40 into the cytosol, however, is not known (Figure 3C, step 4). Because SGTA is an established Hsc70 co-chaperone [84,85], it may regulate Hsc70’s ability to engage SV40. Alternatively, as SGTA exists as a dimer [86], simultaneous binding of a SGTA dimer to one molecule of Hsc70 and one molecule of Hsp105 may bring Hsc70 and Hsp105 in close proximity to facilitate the extraction reaction. Of note, SGTA dimerization is necessary during proper androgen receptor signaling [87]. As one final possibility, since SGTA has been reported to partially localize to the nucleus [88], it may serve a dual role during SV40 infection—in this case, SGTA may promote both SV40 ER-to-cytosol transport as well as cytosol-to-nuclear transport. In addition to SV40 and BK PyV, SGTA also plays a role in other viral infection pathways, such as in HIV [89], parvovirus [90], and severe acute respiratory syndrome coronavirus [91] infections, all of which have structural proteins that interact with SGTA’s central tetratricopeptide (TPR) domain repeats [92]. Because none of these viruses uses the ER-to-cytosol transport pathway for entry, SGTA appears to be exploited by other viruses for different purposes, consistent with its ability to function in numerous cellular pathways [85].

## 4. Conclusions

In this review, we describe in detail a decisive PyV infection step: penetration of the ER membrane that enables the virus to reach the cytosol. Although PyV infections are highly common in the human population and are generally thought to be benign, they can be especially problematic in immunocompromised individuals, such as in AIDS and transplant patients. Thus insights into the molecular basis by which PyVs enter host cells should allow us to develop more effective anti-viral therapies against PyV-induced human diseases including Merkel cell carcinoma, PML, and PyV-induced nephropathy. 

Our analyses revealed that elements of a major ER quality control pathway called ERAD are exploited by the archetype PyV SV40, as well as other PyV family members, during ER membrane penetration. As chemical inhibitors of various components in the ERAD pathway have been reported [93], the ability of these inhibitors to dampen PyV infection should be directly assessed. For instance, ERAD has been implicated in regulation of flavivirus infection [94,95,96,97,98]. Not surprisingly, a recent study identified compounds known to disrupt the Hsc70 chaperone cycle were able to potently block flavivirus infection [93], consistent with the fact that this chaperone cycle controls ERAD. The connection between ERAD and SV40 also has broad implications. For example, the non-enveloped human papillomavirus (HPV) has been reported to traffic to the ER from the cell surface [99], and hijack various PDI family members during infection [100]. Thus it is tempting to speculate that HPV might use aspects of the ERAD machinery to access the cytosol, similar to strategies used by SV40. 

A major gap in our understanding of non-enveloped virus entry is how they breach a host cell membrane to cause disease. For enveloped viruses such as HIV and influenza virus, fusion between the viral and host membranes delivers the core viral particle into the host cytosol [101,102]. By contrast, non-enveloped viruses such as PyV and HPV lack a surrounding membrane, and therefore must reach the cytosol using a fundamentally different mechanism. In the case of SV40, one key principle is that host factors are exploited to create a hydrophobic particle that inserts into the ER membrane, a reaction that initiates the membrane penetration process. Whether this is a general strategy used by other non-enveloped viruses to cross biological membranes is unclear, and is an area of investigation worth pursuing. Another emerging principle is that SV40 appears to trigger formation of a specific membrane penetration site on the ER membrane (called foci) that enables the viral particle to access the cytosol. If true, this would be the first instance of a viral particle that selectively builds a physical structure through which it crosses a host membrane. How this penetration structure is systematically constructed in the ER membrane deserves more attention, and is a question that can be addressed by application of high-resolution microscopy techniques.

In sum, despite emerging insights into the overall PyV infection pathway, many critical questions remain. For instance, how does the virus mobilize to the nucleus after arrival to the cytosol? Is the Hsc70-Hsp105-SGTA complex, known to be crucial during ER-to-cytosol membrane transport, also important during viral nuclear entry? What are the viral structural states in the cytosol and upon entering the nucleus? Addressing these and other critical questions will require further research. These insights will not only prove valuable in illuminating the PyV infection pathway, they should unveil additional therapeutic targets that can be used to better combat the plethora of PyV-induced human diseases.
